# The GC-heterogeneity of teleost fishes

**DOI:** 10.1186/1471-2164-9-632

**Published:** 2008-12-24

**Authors:** Christelle Melodelima, Christian Gautier

**Affiliations:** 1 Laboratoire d'Ecologie Alpine, UMR CNRS 5553, Université J. Fourier, 38041 Grenoble Cedex 9, France; 2 Laboratoire de Biologie et Biométrie Evolutive, CNRS UMR 5558, Claude Bernard University Lyon 1, 69622 Villeurbanne, France

## Abstract

**Background:**

One of the most striking features of mammalian and birds chromosomes is the variation in the guanine-cytosine (GC) content that occurs over scales of hundreds of kilobases to megabases; this is known as the "isochore" structure. Among other vertebrates the presence of isochores depends upon the taxon; isochore are clearly present in Crocodiles and turtles but fish genome seems very homogeneous on GC content. This has suggested a unique isochore origin after the divergence between Sarcopterygii and Actinopterygii, but before that between Sauropsida and mammals. However during more than 30 years of analysis, isochore characteristics have been studied and many important biological properties have been associated with the isochore structure of human genomes. For instance, the genes are more compact and their density is highest in *GC *rich isochores.

**Results:**

This paper shows in teleost fish genomes the existence of "GC segmentation" sharing some of the characteristics of isochores although teleost fish genomes presenting a particular homogeneity in CG content. The entire genomes of *T nigroviridis *and *D rerio *are now available, and this has made it possible to check whether a mosaic structure associated with isochore properties can be found in these fishes. In this study, hidden Markov models were trained on fish genes (*T nigroviridis *and *D rerio*) which were classified by using the isochore class of their human orthologous. A clear segmentation of these genomes was detected.

**Conclusion:**

The GC content is an excellent indicator of isochores in heterogeneous genomes as mammals. The segmentation we obtained were well correlated with GC content and other properties associated to GC content such as gene density, the number of exons per gene and the length of introns. Therefore, the GC content is the main property that allows the detection of isochore but more biological properties have to be taken into account. This method allows detecting isochores in homogeneous genomes.

## Background

The isochore structure refers to the fact that some eukaryotic genomes are organized into mosaics, which are characterized by a having fairly constant average guanine-cytosine (GC) content over scales of kilobases, and then abruptly shifting to another fairly constant-GC-content level [1]. The isochore has been classified as a "fundamental level of genome organization" [2], and this concept has increased our appreciation of the complexity and variability of the composition of eukaryotic genomes [3]. This compositional pattern is typical of vertebrate genomes. However, some authors have identified isochore structure in the *Arabidopsis thaliana *([3-6]). Analyses using density gradient ultracentrifugation have shown that mammal and bird genomes vary widely. In contrast, the genomes of amphibians and fishes (cold-blooded vertebrates) are characterized by a much lower level of compositional heterogeneity [7]. From these observations, a correlation between isochore structure and body temperature pattern is assumed [8]. However, on average, the GC_3 _level of codons is lower in cold-blooded vertebrates than in mammals and chickens, but there is substantial variation in the mean GC_3 _level between cold-blooded vertebrate species ([9-14]). In contrast, even if only partial dataset are available, it seems that almost all cold-blooded vertebrates show substantially less variability in GC_3 _within their genomes than warm-blooded species.

**Figure 1:**
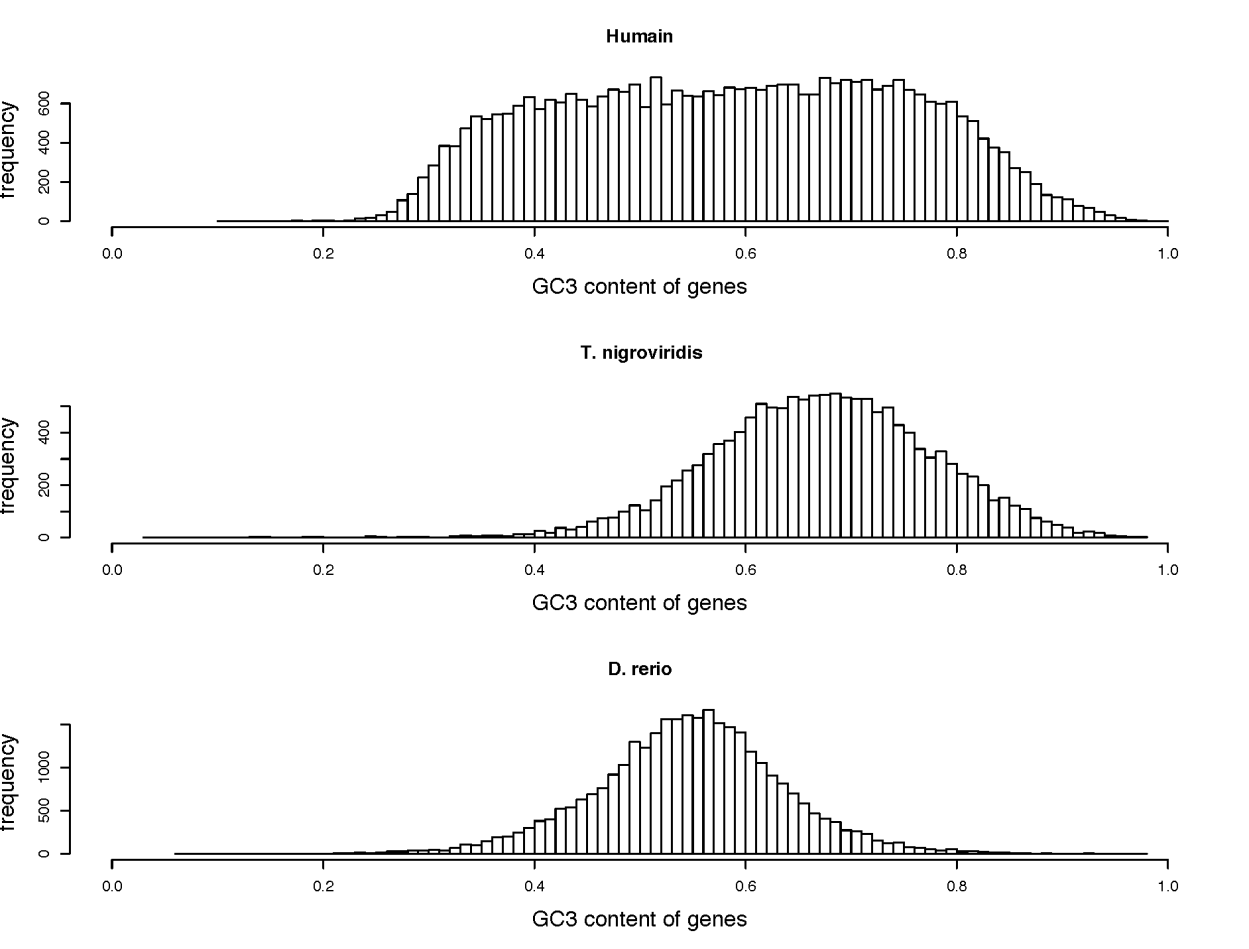
**Distribution of genes according to their GC_3 _content in the human, *T nigroviridis *and *D rerio *genomes**.

The sequencing of the *D rerio *and *T nigroviridis *genomes has made it possible to carry out large scale genome comparisons of fish and other vertebrate genomes, and in particular the human genome. The remarkably compact nature of *the T nigroviridis *genome [15], and the relative homogeneity of the GC content (Figure 1) tend to confirm the absence of isochores in the *T nigroviridis *genome. The *T nigroviridis *and the *D rerio *genome are homogeneous, however, the *D rerio *genome is much longer and its GC content is much lower (Figure 1). Many important biological properties have been associated with the isochore structure of human genomes. In particular, the density of genes has been shown to be higher in GC-rich than in GC poor-isochores ([16,17]). Genes in GC-rich isochores are more compact, with a smaller proportion of intronic sequences, and large proteins are avoided in GC-rich isochores [18]. Additionally, the insertion process of repeated elements depends on the isochore region involved [17]. Therefore, the aim of this study is to investigate whether a mosaic structure associated with isochore properties may be found in these fishes. One of the original features of the present study is that it assumes that for the most part, orthologous genes are found in different species of vertebrate, even if they are as divergent as a fish and a mammal ([15,19]). The characteristics of gene contained in human isochores can be found in fish orthologous genes. Thus, specific Hidden Markov Models (HMMs) were developed to work on fish genes that are orthologous to human genes [20]. Then, the biological properties of segmentation are compared with the biological properties known to be linked to isochores in mammalian genomes. A Moran Index calculated on a sliding window is used to test the quality index of our segmentation.

## Results

### Preliminary results obtained in the chicken genome

In this study, we assume that orthologous genes remain approximately in the same isochore class over evolutionary time between human and fish genomes. Therefore, before assuming it is true in fishes, we have verified that this assumption is true in other isochore-containing genomes. These preliminary studies were conducted on chicken genome since it is more close to the human genome. A correlation between human and chicken GC_3 _values (R = 0.58, p-value < 2.10^-16^) was observed. The mean GC contents were 0.49 (σ = 0.03), 0.44 (σ = 0.03) and 0.40 (σ = 0.02) respectively for the H, M and L isochore classes as defined by our HMM method. The Kruskal-Wallis non-parametric test was significant (p-value < 10^-5^). For all chromosomes, the isochore structure is correlated with the gene density distribution along the chromosome. The gene density in the H isochores (40.2 genes per Mb) was higher than the gene density in the L isochores (15.4 genes per Mb), leading to a significant Wilcoxon test (p-value = 3.10^-8^). The same difference was observed when we compared the characteristics of the M isochores (20.9 genes per Mb) with those of the H (p-value = 5.10–4) and L isochores (p-value = 4.10^-7^). A correlation between the number of exons per gene and isochore class was observed. The number of exons per gene in the H isochore (9) was smaller compared with the number of exons in the isochore L (12.4) (p-value = 2.10^-3^). To conclude, the results of this preliminary study allow us to suppose that characteristics of gene contained in human isochores can be found in fish orthologous genes as in chicken genomes.

### Evaluation of HMMs

**Figure 2:**
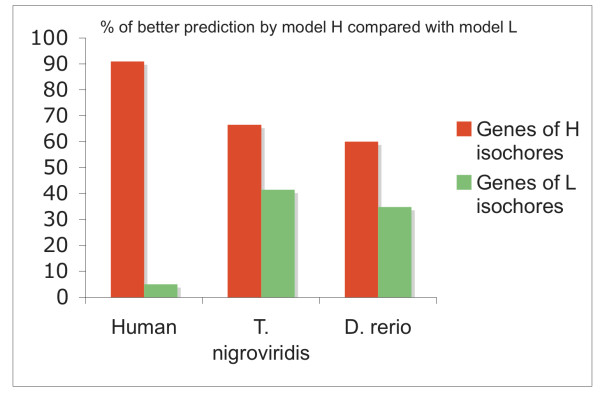
**Prediction of HMM H and L on orthologous genes**. The orthologous genes of test sets H and L of the human, *T nigroviridis *and *D rerio *genomes were compared with the HMM H and L predictions.

Three HMMs, ("H", "M" and "L"), were built. *T nigroviridis *and *D rerio *models "H", "M" and "L" were trained according to the fish genes orthologous to human isochores GC-rich, GC-medium and GC-poor. These HMMs were used to make a segmentation of the genome. The structural differences between genes in the "H" and "L" classes obtained for these two fish species are shown in Figure 2. For each species, the genes belonging to the "H" class were preferentially recognized by the "H" model; whereas the genes of isochore "L" were mainly recognized by the "L" model. The results of the ℵ^2 ^test were highly significant (the p-values were 3.10^-12 ^and 7.10^-9 ^for *T nigroviridis *and *D rerio *respectively). Therefore, and this shows that there is a significant difference in structure between classes "H" and "L" in these two species. If the same approach is used on the set of human genes orthologous to *T nigroviridis *genes a stronger differentiation between *H *and *L *is obtained; the p-value of the test ℵ^2 ^was 2.10^-27^.

**Figure 3:**
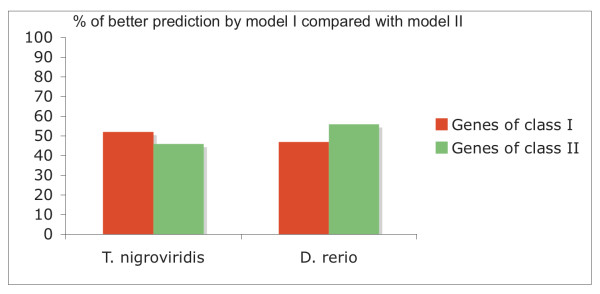
**Prediction of HMM I and II**. Genes of test sets I and II of *T nigroviridis *and *D rerio *genomes were compared with the HMM I and II predictions.

To verify the absence of a methodological bias, genes have been randomly separated into 2 classes (I and II). A Markov model was trained for each of these classes by using a test set containing 2/3 of the genes. Figure 3 shows that these models, as expected, do not discriminated between classes I and II (the p-values of the ℵ^2 ^test were 0.87 and 0.48 for *T nigroviridis *and *D rerio *respectively).

### GC-heterogeneity of Mosaic chromosome maps of the *T nigroviridis *and *D rerio *genomes

**Figure 4:**
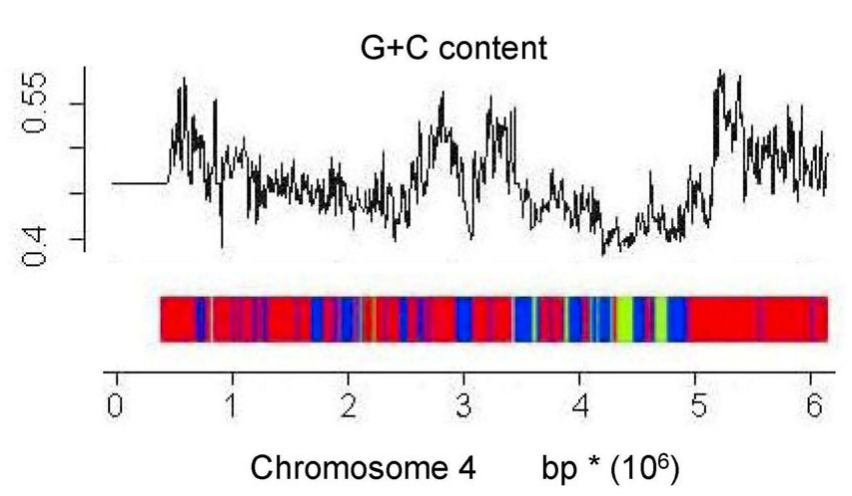
**Distribution of isochores along *T nigroviridis *chromosome 4**. The detected H, L and M isochores are shown in red, green and blue, respectively. To check the consistency of isochore prediction, the graph is shown alongside a plot of the GC content along the chromosome.

**Figure 5:**
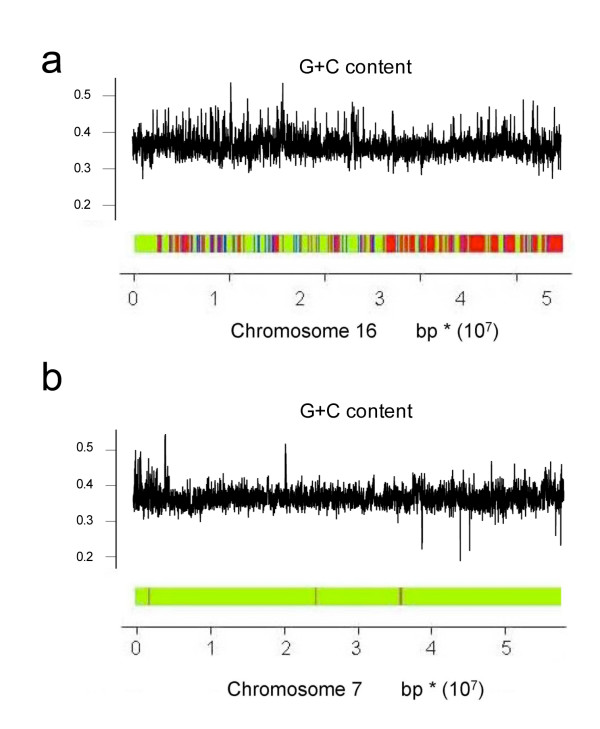
**Distribution of isochores along *D rerio *chromosomes 7 and 16**. The H, L and M isochores detected are shown in red, green and blue respectively. To check the consistency of isochore prediction, the graph is shown alongside a plot of the GC content along the chromosome.

The *T nigroviridis *and *D rerio *genome segmentations obtained by our method are shown in Figures 4 and 5. Maps of all the chromosomes of the *T nigroviridis *and *D rerio *genomes are available online at .

Given the overall GC composition of *T. nigroviridis *(GC-rich) and *D. rerio *(AT-rich), we may suppose that most of the *T. nigroviris *belongs to the H isochore and most of *D. rerio *belongs to the L isochore. Segmentation obtained by your method confirms this hypothesis. Thus, in the *T nigroviridis *genome, most of the isochores belong to class H. The distribution of isochores H and L was fairly similar in the different chromosomes.

The isochores were not uniformly distributed along the chromosomes of the *D rerio *genome. There were more L isochores than H isochores. The main characteristic of the *D rerio *genome was that some chromosomes consisted entirely of L isochores (for example, chromosome 7, Figure 5a), whereas others consisted mainly of H isochores (for example chromosome 16, Figure 5b).

Along the *T nigroviridis *and *D rerio *genomes, the distribution of windows has been compared with 1000 random permutations of the same windows. A significant difference was observed between the average length of isochores obtained by our prediction and the average length of isochores obtained by simulation (the p-values of the ℵ^2 ^test were equal to 2.10^-3 ^for the *T nigroviridis *and 2.10^-15 ^for the *D rerio*, respectively). This test confirms that a regional structure exists in the two fish genomes as consecutive windows have a greater probability to belong to the same isochore than by chance.

### Correlation between biological properties and the GC heterogeneity

The isochore structure of mammalian genomes has been implicated in numerous biological characteristics. We have shown that these characteristics are also linked to the segmentation described here for fish genomes.

#### Size of segmentation

The average length of an isochore depends on the species. On the *T nigroviridis *genome, the H isochores are longer than other types of isochores. The average length for L isochores was 33.1 kb, whereas the average lengths of the M and H isochores were 55.2 kb and 73.1 kb respectively. These lengths were significantly different (Kruskall-Wallis p-value < 10^-8^), however, on the *D rerio *genome, the L isochores were on average longer (638 kb). The lengths of the M and H isochores of *D rerio *were 61.7 kb and 59.4 kb respectively. These lengths were significantly different (Kruskall-Wallis p-value < 10^-12^).

**Figure 6:**
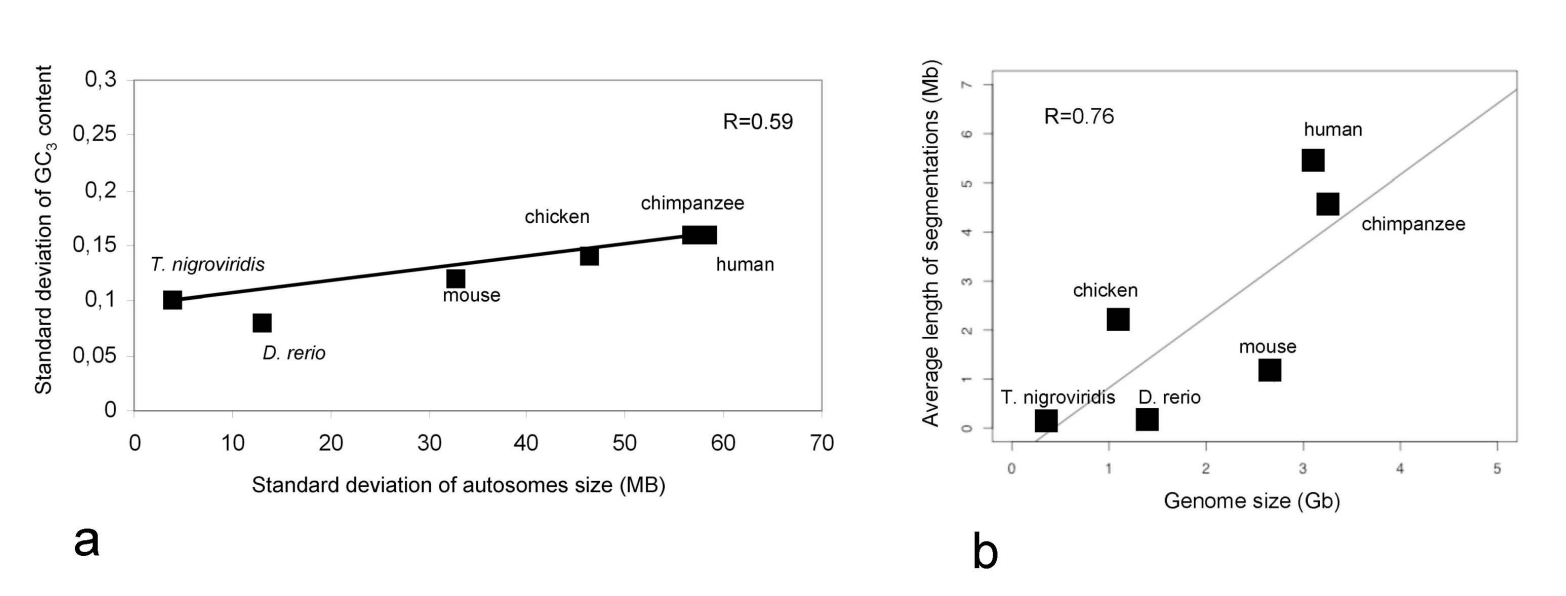
**Size of segmentation and length of genome**. a) Correlation between the size of segmentation and the length of the genome. b) Variability of the size of autosomes and the GC3 content.

There is a correlation between the size of segmentation and the length of the genome. This has been studied for *T. nigroviridis*, *D. rerio*, chicken, human, chimpanzee and mouse (Figure 6a). The correlation value was R = 0.76. Moreover, the variability of the size of the autosomes was linked to the variability of the GC content (R = 0.59) (figure 6b).

#### GC content of each type of segmentation

**Figure 7:**
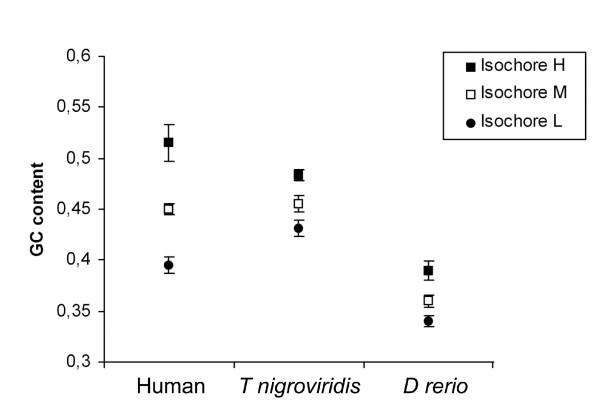
**GC contents for H, M and L isochore predicted by our HMM in vertebrates**. The human, mouse, *T nigroviridis *and *D rerio *genome were analyzed. For each species, the Kruskal and Wallis non-parametric test comparing the GC content of the different classes of isochore was significant (p-values were equal 10^-8^, 5.10^-4 ^and 2. 10^-3 ^for human, *T nigroviridis *and *D rerio *species respectively).

**Figure 8:**
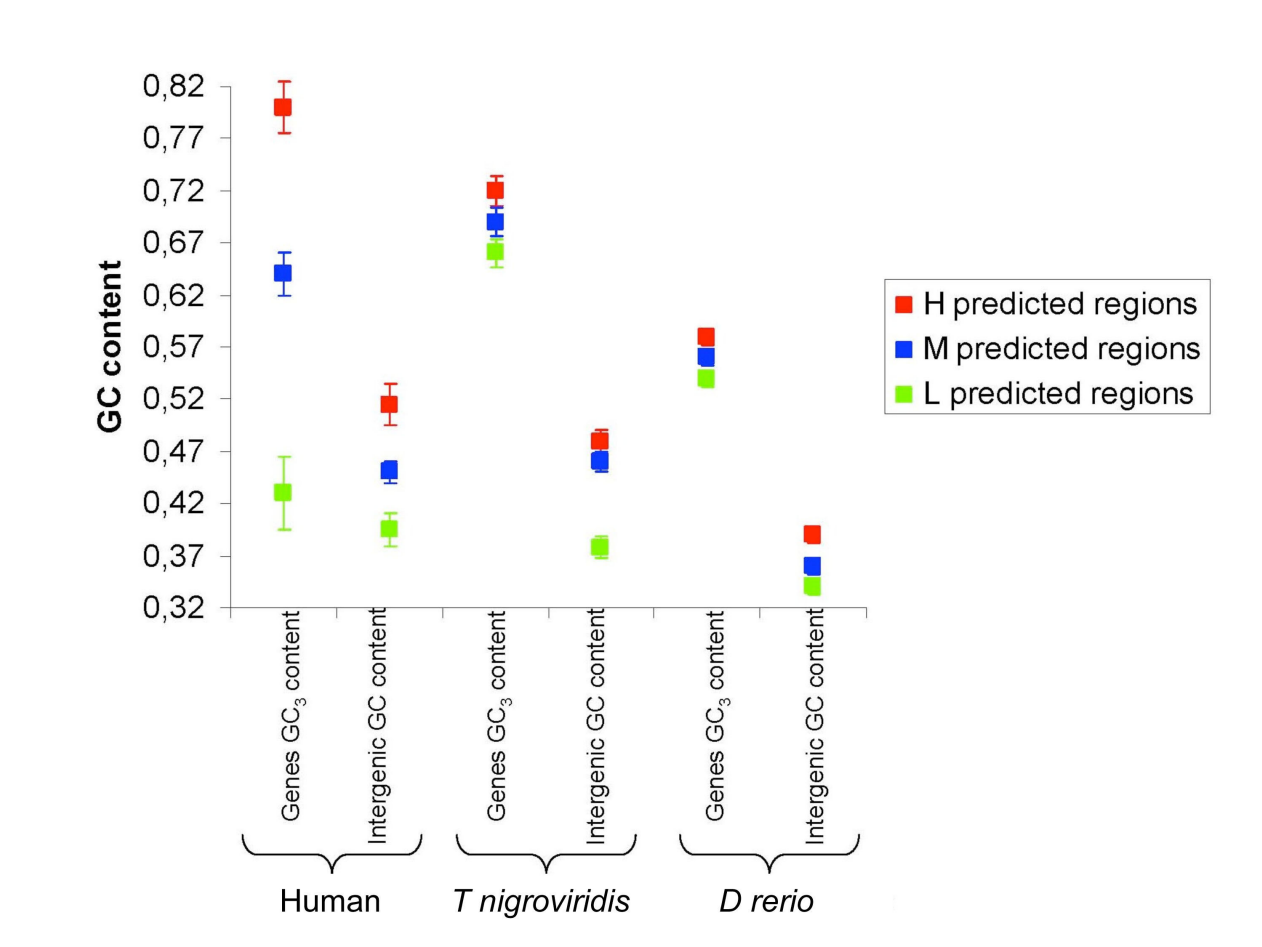
**GC contents of intergenic regions and GC_3 _content of CDS contained respectively in H, M and L isochore predicted in vertebrates by our HMMs**.

The GC content for *H*, *M *and *L *isochores from the two Teleost fishes and human (from [17]) are shown in figure 7 and 8. The *D rerio *genome is more homogeneous compared with the *T nigroviridis *genome but the segmentation of the two fish genomes was related to the GC content.

#### Gene distribution in each type of segmentation

The percentage of the coding region in each isochore class was consistent with that found for mammalian genomes [16]. For the *T nigroviridis*, the coding regions correspond to 10.2% of the H isochores, and 5.5% of the L isochores. The p-value of the Wilcoxon test was significant (p = 2.10^-3^). For *D rerio *genome, the coding regions correspond to 1.8% of the H isochores, and 1.3% of the L isochores (p-value = 3.10^-2^).

#### Transposable elements

In the human genome, the insertion process of repeated elements depends on the isochore region involved [17]. We have investigated the correlation between transposable elements and mosaic segmentations along *T. nigroviridis *and *D. rerio*. No effect of repeats has been observed on our segmentation of *T. nigroviridis *and *D. rerio*.

### Gene structure in each type of segmentation

The length distributions of exons were approximately the same in the three isochore classes for all three species (Table 1). However, initial exons tended to be longer in the H classes of these three species. Human and *D rerio *introns were longer than *T nigroviridis *introns. Furthermore, human and *D rerio *intron lengths depend on the isochore class, whereas this is not true for the *T nigroviridis*. The number of exons per gene was similar in the two fish species (Table 2). A correlation between the number of exons per gene and per isochore class was observed for each species. Finally, the GC content of exons and introns vary significantly in the human and *T nigroviridis *genomes depending on the isochore class, but was only significant for CDS in the case of *D rerio *(Table 3).

**Table 1:** Length of exons and introns in the human, *D rerio *and *T nigroviridis *genomes

Position in genes	Length in class H (average bp)			Length in class M (average bp)			Length in class L (average bp)		
	
	human	*T nigroviridis*	*D rerio*	human	*T nigroviridis*	*D rerio*	human	*T nigroviridis*	*D rerio*
Initial coding exon	233	205	211	176	198	206	160	167	190
Internal exon	144	156	144	143	159	150	144	151	144
Terminal Exon	244	253	204	237	253	200	218	233	189
Intron	1275	594	1350	1809	597	1578	3117	606	1830

Only introns localized between two coding exons were considered.

**Table 2:** Comparison of the number of exons between human, *D rerio *and *T nigroviridis*

Predicted regions	Number of exons (average)		
	
	human	*T nigroviridis*	*D rerio*
*H*	8.93	8.31	8.9
*M*	10.76	9.94	9.8
*L*	12.31	11.03	10.79

**Table 3:** Comparison of GC content between human, *T nigroviridis *and *D rerio*.

Region of genes	Predicted regions	human	*T nigroviridis*	*D rerio*
GC_3 _of CDS	H	0.8(σ = 5.10^-2^)	0.72(σ = 9.10^-3^)	0.58(σ = 6.10^-3^)
	M	0.64(σ = 4.10^-2^)	0.69(σ = 9.10^-3^)	0.56(σ = 6.10^-3^)
	L	0.43(σ = 7.10^-2^)	0.66(σ = 9.10^-3^)	0.54(σ = 6.10^-3^)
	Kruskal-Wallis p-value	2.10^-16^	8.10^-3^	5.10^-2^
	Δ (H-L)	0.37	0.06	0.04

GC of introns	H	0.59(σ = 9.10^-2^)	0.48(σ = 6.10^-2^)	0.349(σ = 6.10^-2^)
	M	0.51(σ = 9.10^-2^)	0.44(σ = 5.10^-2^)	0.346(σ = 5.10^-2^)
	L	0.38(σ = 9.10^-2^)	0.41(σ = 6.10^-2^)	0.345(σ = 6.10^-2^)
	Kruskal-Wallis p-value	3.10^-5^	2. 10^-3^	0.7
	Δ (H-L)	0.21	0.07	0.004

### Influence of the GC content on segmentations

The 7753 pairs of orthologous genes used to train the model were used to compare the GC_3 _content in the three classes. As expected, the Kruskal-Wallis test was highly significant (p-value < 2.2 10^-16^) for human genes, and the difference was also significant (p-value = 8 10^-3^) for the *T nigroviridis *genes. The difference in the GC_3 _content was preserved between the two species, although this difference was clearly weaker in the *T nigroviridis *genome than in the Human genome. To evaluate the role of GC_3 _content in our *T nigroviridis *genome segmentation, a new model based on three classes defined by their GC_3 _content was built. Three classes were defined based on the GC frequencies at the third codon position (GC_3_). The limits were set so that all three classes contained approximately the same number of genes. This yielded classes HGC = [100%, 75%], MGC = ]61%,75% [and LGC = [0%,61%]. Two thirds of the genes were used to train HMM models, and the remaining genes were used for testing. For *T nigroviridis*, the likelihood of LGC and HGC Markov models revealed a significant difference between the LGC and HGC classes (the p-value of the ℵ^2 ^test was equal to 6.10^-11^). However, the difference between the LGC and HGC classes was not as great as that between the "H" and "L" classes, and the p-values were 6.10^-11 ^and 3.10^-12 ^respectively. Comparing the genes in class HGC to those in class H showed that only 57% were the same. In the MGC and LGC classes, only 60% and 58% of genes respectively were the same as those in classes M and L.

The same study was carried out on *D rerio *genes. In this case, the comparison of the GC_3 _content in the three classes by the Kruskal-Wallis test was weakly significant (p-value 5.10^-2^). For *D rerio*, the following limits were used for the classes: HGC = [100%, 61%], MGC = ]56%,61% [and LGC = [0%,56%]. A comparison of LGC and HGC reveals a significant difference (the p-value of the ℵ^2 ^test was equal to 3.10^-4^). However, the difference between classes LGC and HGC was less marked than between classes "H" and "L", the p-values were 3.10^-4 ^and 7.10^-9 ^respectively.

### Analysis of the spatial structure along the *T nigroviridis *and *D rerio *genomes

The existence of an organizational structure linked to the distribution of the GC_3 _and the GC content along the *T nigroviridis *and *D rerio *genomes was analyzed by computing: (i) for each chromosome, the Moran's Index based on the GC_3 _of genes distributed along the chromosome and (ii) the Moran's Index based on the GC content (windows of 14 kb were used) (Table 4). For the two fishes, these tests show a high autocorrelation of GC, and a clear but weaker autocorrelation of GC_3_. Autocorrelations were higher in *T nigroviridis *than in *D rerio*.

**Table 4:** Moran Index

	*T nigroviridis*			*D rerio*		
	
	Minimum	Maximum	p-value	Minimum	Maximum	p-value
GC_3_	0.15	0.35	<10^-5^	0.28	0.47	<10^-6^
GC	0.8	0.95	<10^-16^	0.43	0.92	<10^-16^
*P [H | W]*	0.65	0.79	<10^-16^	0.45	0.67	<10^-6^
*P [L | W]*	0.47	0.6	<10^-5^	0.52	0.76	<10^-16^

For all *T nigroviridis *and D chromosomes, maximum and minimum values of the Moran Index in the four cases of autocorrelation (GC3, GC, *P [H | W] *and P *[L | W], W = window of 14 kb*). In all cases, the Moran test was highly significant and confirmed the presence of a spatial organization.

To quantify the level of segmentation obtained by our method, we computed for each chromosome (i) the Moran's Index based on the *P [H | W] *of windows distributed along the chromosome and (ii) the Moran's Index according to the *P [L | W] *of windows distributed along the chromosome. For the *T nigroviridis *genome, a high autocorrelation of the *P [H | W] *was found. The autocorrelation of the *P [L | W] *was less obvious, but still significant. For the *D rerio *genome, the opposite was observed: the autocorrelation of the *P [L | W] *was clearer than the autocorrelation of the *P [H | W]*, however correlations were significant in all cases.

## Discussion

The existence of clustering of high-GC and low-GC regions within the genomes of mammals and birds is generally accepted. Recently, some authors have shown the presence of isochore structures in the Arabidopsis Thaliana ([3,6]), or in Apis mellifera ([21]). These studies tend to show that regional compositional structures are not random and/or restricted to specific taxa as vertebrate. Additionally, we in the present study and in a previous one about human genome [20] we have shown that segmentations were linked to several biological properties (gene density, the number of exons per gene and the length of introns) and can not be considered as random sequences.

The originality of our approach was that it assumed that the characteristics of genes contained in human isochores would also be found in orthologous genes of species not thought to have isochores. Therefore, the GC content was not the only feature we used to segment fish genomes. A difference in the quality of the predictions of models between human and fishes has been identified in this study. To construct the H and L classes of fish, we have assumed that each fish gene has kept at least one characteristic related to the isochore class of the orthologous human gene. However, some genes could have lost this characteristic as a result of evolving differently in the two species. Therefore, the difference in prediction accuracy between human and fish could be explained by the presence of these genes since their GC content is different compared with the GC of isochore class in fish. Nevertheless, although the mammals and fishes separated more than 450 million years ago, we have found a correlation between human and Tetraodon GC_3 _values (R = 0.25, p-value < 2.10^-16^) as well as between human and D. rerio GC3 (R = 0.19, p-value < 2.10^-16^). Thus, in species thought not to have isochores, it was possible to find signs of isochores derived from the orthologous mammalian genes. No limits of the GC content of the isochore classes were fixed, but they varied from one species to another according to the content and the homogeneity of the GC of the genome studied. Many regions associated with human isochores could be characterized and predicted thanks to factors other than the GC content, such as the intron lengths and the gene density.

The segmentations obtained in this paper were linked to isochores properties of mammal genomes (size of segmentation, GC content, gene density, and gene structure). There was a significant difference between the isochore distribution found using our method of prediction windows, and a random distribution of these windows. There were more coding regions in the H isochore than in the L isochore in both these fish species. However, the difference between the ratio of coding regions in isochores H and L was weaker in the *D rerio *genome than in the *T nigroviridis *genome. The segmentation obtained for the *D rerio *genome was longer compared with the segmentation observed in *T nigroviridis *genome. In *D rerio*, some chromosomes had only L isochores, whereas others contained only H isochores. Nevertheless, the distribution of genes between the different chromosomes was approximately the same.

Pizon et al. [22] have suggested that at least two families of isochores were found for a tetradontid fish. Moreover, the Moran's Index for the GC_3 _content, the GC content and the probability values *P [H | S] *and *P [L | S] *show that our segmentation has a link with the GC content. These results show that the use of characteristics associated to the isochore organization that are complementary of the GC content, for example gene density or gene structure, may improve the detection of isochores.

Furthermore, the comparison of the performance of the hidden Markov models adapted to the "H", "L" and "M" classes with those adapted to random classes reinforce the idea that the characteristics of gene depend of their isochore class. This is more than a simple "isochore" map of the fish genomes. The training of the HMMs ("*H*", "*M*", "*L*"), and their comparison using test sets show some differences of characteristics between the genes *T nigroviridis *and those of *D rerio*. For example, gene density, the length of the initial exons and the length of introns were different for genes classified as belonging to "H", and belonging to "the predicted H isochore", than those for genes classified as "L" and belonging to the predicted L isochore.

## Conclusion

The genomes of mammals and birds are mosaics of isochores, i.e. long DNA segments relatively homogeneous in GC content when compared to the pronounced heterogeneity throughout the entire genome. The present study reveals that there is a mosaic structure related to isochores in the genomes of both *T nigroviridis *and *D rerio *although they are characterized by lower level of compositional heterogeneity. Thus, the homogeneity of the GC content of isochores should be considered to be relative. In conclusion, an updated definition of isochore can be proposed since isochore can be detected also in compositionally homogeneous genomes. Isochores are a segment of genome DNA, in which many characteristics, such as gene density, GC content, the number of exons per gene and the length of introns are different from one isochores to another.

## Methods

### Materials

For the preliminary study, the orthologous Human and chicken genes were extracted from GemCore . Pairs of orthologous genes have been inferred by reciprocal best hit from sequences in ENSEMBL. This approach is quicker than phylogenetic analysis, and gives similar results once whole genomes have been established. This procedure yielded a set of 6821 orthologous genes between human and chicken genomes.

Similarly, the orthologous Human and *T nigroviridis *genes were extracted from GemCore. Pairs of orthologous genes have been inferred by reciprocal best hit from sequences in ENSEMBL. This procedure yielded a set of orthologous 7753 genes between Human and *T nigroviridis*. These genes corresponded to 27% of all the genes annotated by Ensembl. Similarly, 8872 human and *D rerio *orthologous genes were extracted. Data on all *T nigroviridis *and *D rerio *chromosomes were retrieved from Ensembl. These data were used to train HMMs. The segmentations and their analysis have been performed on the entire genomes of the *T nigroviridis *and *D rerio*.

### Mosaic chromosome maps of the *T nigroviridis *and *D rerio *genomes

Based on the work realized for the human genome[20], HMMs have been built, adapted and trained on *T nigroviridis *and *D rerio *genes. High, Medium and Low-density genomic segments are known as H, M and L isochores respectively, in order of decreasing GC content. Four steps are required to locate isochores along the *T nigroviridis *and the *D rerio *genomes:

#### • Model learning procedures

The three isochore regions (H, L and M) of the *T nigroviridis *and *D rerio *genomes were characterized by three HMMs ("*H*", "*M*" and "*L*"). Each region (intergenic, intronic or exonic) was taken into account, and represented by a macro-state in each of the HMMs, H, M and L. In addition, exons consist of a succession of codons. Each of the three possible positions in a codon (1, 2, 3) has its own characteristic statistical properties, and was taken into account. Additionally, each HMM also takes into account the direct and reverse strands of the DNA sequences [17]. *T nigroviridis *and *D rerio *genes were used to train. *nigroviridis *and *D rerio *models. Fish genes corresponding to their orthologous genes located in the H, M and L isochores of the human genome were selected as belonging to the H, M and L isochores of fishes. The H, M and L classes contained 2304, 2134 and 3314 genes respectively in the *T nigroviridis *genome, and 2619, 2437 and 3816 genes in the *D rerio *genome. To constitute a training set and a test set, genes of each class were randomly separated. The training and test sets contained 2/3 and 1/3 of the genes respectively. This distribution provided enough data to train the models, and also to obtain a significant number of genes to test the efficiency of the models. Lastly, a hidden Markov model was adapted to each isochore class [20].

#### • Sliding windows

the DNA of each chromosome was divided into 14 kb overlapping windows. Two successive windows overlapped by half their length. These windows were smaller that those in the study conducted on the human genome since the *T nigroviridis *and *D rerio *genomes were smaller than the human genome [20]. The compact nature of the *T nigroviridis *genome suggests that these windows may contain genes. This was important, because the gene unit was the principal discriminating information for the predictions of our HMM.

#### • Segmentation by a Bayesian approach

For each window and for each model (*H*,*L *and *M*), the probability *P [Mod | S] *was obtained using equation 1:

(1) $$P(Mod|S)=\frac{P(S|Mod)\times P(Mod)}{\sum_{m\in \left\{H,M,L\right\}}P(S|m)\times P(m)},$$

where *Mod *is "*H"*,*"L" *or "*M"*, and *S *the window that is being tested,*P(S|Mod) *was computed by the forward algorithm using the SARMENT package [23]. In our case, the characteristics of *P(Mod) *were unknown. We estimated them as P(*H*) ≈ P(*M*) ≈ P(*L*) ≈ 1/3. As a consequence, our Bayesian approach was numerically very close to a maximum likelihood approach. The model with the best probability characterizes the isochore type allocated to a window. The segmentation is represented by this succession of windows.

### Mosaic chromosome of chicken genome

To confirm results obtained on fish genome, it was interesting to test if orthologous genes remain approximately in the same isochore class over evolutionary time in some other isochore containing genome. Thus, the procedure described before has been applied on chicken genome because human and chicken genomes are closer than human and fish genomes.

#### • Evaluation of our segmentation

Several tests were performed in order to check the consistency of the isochore prediction: (i) the distribution of isochores was plotted versus the GC content along the chromosome; (ii) the ratio of coding regions was compared between the H and L isochores predicted by our method; (iii) furthermore, the segmentation made it possible to define the isochore class of each window along the genomes of *T nigroviridis *and *D rerio*. The distribution of isochores in these windows was compared to a random distribution of these windows. One thousand simulations were carried out.

### Evaluation of HMMs

A supplementary analysis was carried out in order to check that various different structures had been preserved in the *T nigroviridis *and *D rerio *genes according to the isochore classes (H, L and M) of their orthologous genes in the human genome. Two analyses were performed:

#### Test sets

The predictions of the *H *and *L *models were compared to the H and L gene test sets in order to determine the degree of differentiation between these two classes of genes.

#### Random sets

The *H *and *L *models were compared to random models. For each fish species, the set of orthologous genes was split randomly into two sets corresponding to two new classes: I and II. Each of these classes contained one half of the orthologous genes. Each class was then split randomly into a training set and a test set, 2/3 of genes were attributed to the training sets. Two models were trained using training sets I and II. The predictions of models I and II were compared to the test sets I and II.

### Analysis of the spatial structure along the *T nigroviridis *and *D rerio *genome

We proposed to use a Moran Index calculated on a sliding window as the quality index. The Moran index is a correlation coefficient, and is used to estimate the degree of spatial autocorrelation at all windows. The Moran Index is given by the ratio of the covariance over the variance as shown in equation 2:

(2) $$I=\frac{n}{\sum_{ij}w_{i,j}}\frac{\sum_{i,j}w_{ij}(x_{i}-\bar{x})(x_{j}-\bar{x})}{\sum_{i}{\left(x_{i}-\bar{x}\right)}^{2}}$$

In this study the Moran's Index was used for each chromosome in order to measure the autocorrelation of (i) the spatial GC_3 _distribution, (ii) the spatial GC distribution, and (iii) the spatial autocorrelation of the prediction of models H and L: *P [H | S] *et *P [L | S]*.

## Authors' contributions

CM carried the statistical analysis, was in charge of writing the codes and the programming aspects of the paper and drafted the manuscript. CM and GC conceived and coordinated the study. CM and GC participated in the design of the study. Both authors read and approved the final manuscript.
